# Estimated intelligence quotient in anorexia nervosa: a systematic review and meta-analysis of the literature

**DOI:** 10.1186/1744-859X-9-40

**Published:** 2010-12-23

**Authors:** Carolina Lopez, Daniel Stahl, Kate Tchanturia

**Affiliations:** 1 Institute of Psychiatry, Kings College London, London, UK; 2 Faculty of Medicine, University of Chile, Santiago, Chile

## Abstract

**Background:**

It has been hypothesised that people with anorexia nervosa have a higher intelligence quotient (IQ) level than the general population. The purpose of this review was to systematically appraise the research into reported IQ levels in people with anorexia nervosa.

**Methods:**

A search using the terms intelligence quotient, IQ, intelligence, cognition, eating disorders and anorexia was conducted in electronic databases only.

**Results:**

In all, 30 peer-reviewed studies written in English that used well established measures of intelligence quotient (the National Adult Reading Test and Wechsler Intelligence Scales) were identified. This review established that people with anorexia nervosa score 10.8 units and 5.9 units above the average intelligence quotient of the normative population on the National Adult Reading Test and Wechsler Intelligence Scales, respectively. An association was found between Body Mass Index and intelligence quotient, as measured by the National Adult Reading Test.

**Conclusions:**

More studies including other eating disorder categories and recovered people are needed to explore important questions regarding the role of the intelligence quotient in treatment response.

## Introduction

Eating disorders (EDs) are a group of psychiatric disorders with a lifelong course and considerable morbidity and mortality. In the Diagnostic and Statistical Manual of Mental Disorders, fourth edition (DSM-IV), EDs include anorexia nervosa (AN), bulimia nervosa (BN), and eating disorders not otherwise specified (EDNOS). The aetiology of EDs remains unknown.

There has been increasing interest in the study of the neuropsychological functioning of people with EDs for the last three decades. One of the reasons for this is to gain a better understanding of the aetiology and maintenance of these disorders and to explore ways of improving available treatments [1].

Although there are more than 100 papers on neuropsychology and brain imaging in EDs, in previous systematic reviews conducted by Roberts *et al. *[2] and Lopez *et al. *[3] on set shifting and central coherence in EDs, it was demonstrated that there are a limited number of neuropsychological studies on BN and those recovered from the disorder. Most studies have explored neuropsychological functioning in AN. Because there is more available research in neuropsychology in AN, the present systematic review focused on AN only.

AN is the most severe ED affecting mainly, but not only, young women, and has the highest rate of mortality linked to a psychiatric disorder, due to high levels of medical complications and suicide in chronic patients [4].

Currently, psychological therapy is the main therapeutic intervention recommended for the treatment of AN (see, for example, [5]). However, outcomes are far from satisfactory with only 50% of cases reaching recovery in adult populations [4,6]. It is possible that factors such as cognitive functioning, usually limited during the acute phase of AN [7], influence treatment utilisation and outcome. For instance, most available therapies require the patient to have some level of insight and verbal ability; factors that might be related to optimal intellectual level [8].

The intelligence quotient (IQ) represents a composite score on a variety of tests designed to measure a hypothesised general ability or intelligence [9]. It has been hypothesised that people with AN have a higher IQ level than the general population based on clinical and school performance observations. There is a suggestion, however, that higher perfectionism, but not higher IQ, would explain the better performance at school in this group [10,11].

As neuropsychological studies have become more popular in AN, there are several studies that have measured intelligence as part of their assessments. However, there are a limited number of studies looking specifically at IQ levels in people with AN. We were not able to find any systematic review summarising research on intelligence in AN. Therefore, the question about IQ in AN remains unanswered. It is expected that examining the available literature in this area would be helpful in providing information about intellectual functioning in AN, investigating how comparable neuropsychological studies from different academic groups are in the context of IQ estimation, helping to clarify what the most appropriate IQ measure would be for future studies, and exploring any association between severity of illness (measured by Body Mass Index (BMI)) and IQ levels.

With these questions in mind, a systematic review of the literature and meta-analysis with the available data on IQ in AN were conducted to address the hypothesis that people with AN show superior scores on well validated IQ tests in comparison with the average IQ of the normative population (norm). This hypothesis is based on the common but conflicting assumption that high intelligence is a trait among people with AN [12,13].

We have also predicted that people with a past history of AN, now recovered, would demonstrate higher IQ scores than those who are in the acute phase of AN and the normative population. We based this hypothesis on the fact that it was shown that IQ predicts termination from treatment; that is, patients with a higher IQ are more likely to remain in psychological treatment [14].

## Methods

### Search procedure and data extraction

This review follows the Preferred Reporting Items for Systematic reviews and Meta-Analyses (PRISMA) statement for meta-analysis [15]. The following electronic databases were used to identify relevant papers for inclusion in this review: Medline, Embase, Psych Info and ISI Web of Science. A first search was conducted in September 2008 and subsequently updated in March 2009.

A broad search was first run on the literature using the terms 'intelligence quotient', 'IQ', 'intelligence', 'neuropsychological assessment', 'neuropsychology', 'cognition', and 'eating disorders' (including 'anorexia', 'bulimia', 'EDNOS', 'recovered anorexia', 'recovered bulimia'). After failing to obtain a substantial number of studies in bulimia nervosa (BN) or recovered AN that included estimated IQ data this search was narrowed to studies that included samples with AN only.

In this manner, the search was conducted by two independent researchers searching for published studies on the basis of the following inclusion criteria:

• Participants. Studies including subjects diagnosed with AN and a sample greater than 10 participants.

• IQ measures. Studies using well known measures to estimate IQ. Specifically, studies including the National Adult Reading Test (NART) and Wechsler scales (Wechsler Adult Intelligence Scale (WAIS), Wechsler Intelligence Scale for Children (WISC), short version of the WAIS (Wechsler Abbreviated Scale of Intelligence; WASI) and the German version of WISC (Hamburg-Wechsler-Intelligenztest für Kinder; HAWIK).

• Data. Studies reporting at least full scale IQ data.

• Language. Studies published in English.

Results from these searches were merged for higher reliability. Following the initial identification of relevant published articles, all citations were then obtained. Further relevant references cited in the retrieved papers were pursued.

### Instruments

The NART [16] is a word-reading test (50 short words of irregular pronunciation) widely used in research and clinical practice as an estimate of premorbid intellectual ability [17]. It has high construct validity as a measure of general intelligence and high levels of inter-rater and test-retest reliability.

The Wechsler Intelligence Scales provide a current estimation of IQ in the adult (WAIS) and child (WISC) versions. These scales are composed of comprehensive intellectual batteries that alternate verbal with visual-perceptual or construction (performance) tests as standard procedure. The full scale IQ test is broken down into subscales comprising verbal (information, digit span, vocabulary, arithmetic, comprehension, similarities) and performance scales (picture completion, picture arrangement, block design, object assembly and digit symbol).

It has been shown that NART and WAIS performance correlate strongly [18]. For instance, correlations between the NART IQ estimates and the WAIS and revised WAIS (WAIS-R) British version are in the range of 0.72 [9].

In both measures, for every age group, a norm of 100 corresponds to the average and 15 to the standard deviation. This permits direct comparison between individual scores with the normative data from the same age range.

### Data synthesis

Meta-analyses were carried out using Stata V. 9.1 (Stata, College Station, TX, USA) using the user-contributed commands for meta-analyses: 'metan' [19], 'metainf' [20], 'metabias' [21] and 'metatrim' [22].

In order to estimate whether the IQ of those suffering from AN differs from the normative population, the data provided by each study was compared with the mean and SD from the normative population, known to be a mean of 100 and standard deviation of 15 (Lezak et al. [9]). The differences in mean IQ scores were standardised by dividing the difference of IQ of patients with an eating disorder and the norm group by the standard deviation of the norm group [23], which is equivalent to Cohen's or Glass's d. The standard error of the effect size was calculated by $SE(d)=\frac{1}{\sqrt{n_{patient}}}$.

Because the mean and the standard deviation of the norm group are regarded as known (based on a large sample size), a bias correction of the standard error is not necessary. The effect sizes and standard errors of the studies were then pooled using random-effect models, which allowed us to model possible study-to-study variation of effect sizes [24].

Meta-analyses were preformed separately for each of the two instruments (NART and Wechsler's derived tests) using the user-contributed Stata command 'metan'. The standardised effect sizes were also back transformed into IQ score differences.

**Figure 1:**
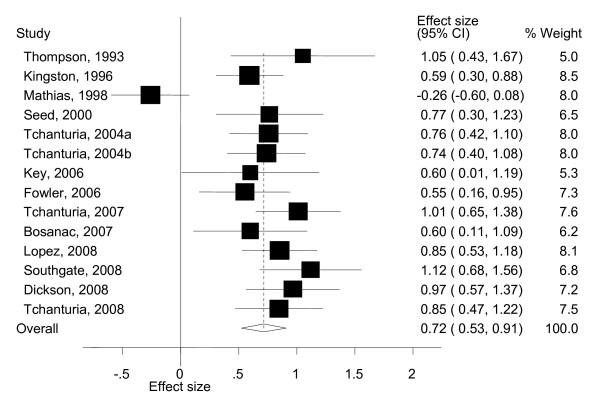
**Forest plot for intelligence quotient (IQ) studies using the National Adult Reading Test (NART): standardised effects for patients with eating disorders (EDs) relative to the normative population (norm) group**.

**Figure 2:**
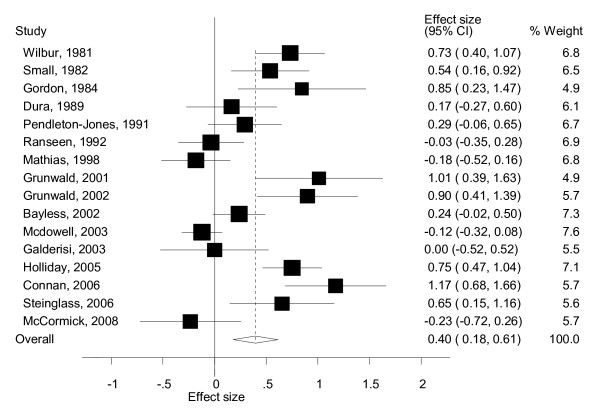
**Forest plot for intelligence quotient (IQ) studies using Wechsler's tests: standardised effects for patients with eating disorders (EDs) relative to the normative population (norm) group**.

The results of the meta-analyses are reported as Forest plots. Forest plots display the results of the meta-analyses in graphical format (see Figures 1 and 2). These graphs represent the variation between the results of the various studies and an estimate of the overall effect size of all the studies together considering the data available for each study included in the meta-analysis [25]. Each line of the Forest plot represents an individual study/comparison. The position of the square in relation to the vertical axis represents the point estimate of the results of a particular study; specifically it shows how the effect size of the study varies from zero. The size of the square shows the weighed individual contribution of the study to the meta-analysis and it is proportional to the sample size of the study. The horizontal line through the square represents the 95% confidence interval (CI) of the effect size. The overall estimate from the meta-analysis and its CI are displayed at the bottom of the plot, represented as a diamond.

Homogeneity between the trials was analysed using Cochran's Q test. Due to the small sample sizes, an additional measure of heterogeneity or inconsistency I^2 ^(Q-(df)/Q) was calculated [26]. I^2 ^describes the percentage of total variation across studies that is due to heterogeneity rather than chance and ranges between 0% (no inconsistency) and 100% (high heterogeneity), with values of 25%, 50% and 75% suggested as low, moderate and high heterogeneity [26].

The analyses were repeated excluding one study at a time to investigate the influence of each individual study on the overall meta-analysis summary using Stata's user-written function 'metainf'.

Statistically significant results are more likely to be published than studies with non-significant results. Therefore, the presence of publication bias was assessed informally by visual inspections of funnel plots, which represent a plot of a study's precision (1/standard error) against effect size. The absence of studies in the right bottom corner (low precision and small effect sizes) of a funnel plot is usually taken as an indication of publication bias. The visual assessments were corroborated by its corresponding statistical analogue, Begg's adjusted rank test [27], and additionally by Egger's test [28], as implemented in 'metabias'.

We also used the Duval and Tweedie [29] non-parametric 'trim and fill' method of accounting for publication bias in meta-analysis as implemented in Stata's user-written command 'metatrim' [30]. If the conclusion of the meta-analysis remains unchanged following adjustment for the publication bias using the trim and fill method, the results can be considered as robust, excluding publication bias.

## Results

After careful assessment on a case-by-case basis, 30 studies (including 849 AN patients in total) were found using the above-mentioned criteria. All of the identified articles used either the NART (N = 14) or Wechsler's derived tests (WAIS, WISC, short forms of WAIS and HAWIK tests) (N = 16).

### Sources of participants

All the selected studies used IQ test on people with AN or recovered. In most studies participants came from clinical populations (N = 28). In all, 14 studies involved only participants who were inpatients at the time of the study, 2 involved outpatients only, 8 involved both inpatients and outpatients, 4 included community samples apart from inpatients and outpatients and 1 study stated that their sample came from a volunteer database only [31]. One study did not specify the source of their participants.

Table 1 shows details of the studies included in this review. All the estimated IQ data is summarised in the meta-analyses described below.

**Table 1:** Studies included in this review

Lead authors	Publication date and reference	IQ test	N	Age	BMI	IQ mean	IQ SD
NART studies:							
Kingston	1996 [41]	NART	46	22.1	14.7	108.9	5.7
Mathias	1998 [32]	NART-R	34	22	15.3	96.1	8.8
Seed	2000 [42]	NART	18	27.3	15.2	111.5	9.4
Tchanturia	2004 [43]	NART-R	34	26.7	13.7	111.4	6.5
Tchanturia	2004 [37]	NART-R	34	27.2	13.7	111.1	7
Fowler	2006 [44]	NART	25	16.9	15.3	108.3	5.5
Tchanturia	2007 [36]	NART	29	28.5	15.5	115.2	5.5
Bosanac	2007 [35]	NART	16	28.9	15.2	109	8.6
Lopez	2008 [45]	NART	37	28.4	15.8	112.8	6.8
Southgate	2008 [46]	NART	20	26.8	16.3	116.8	4.8
Dickson	2008 [31]	NART	24	30.6	16	114.5	5
Tchanturia	2008 [47]	NART	27	28.8	14.3	112.7	6.5
Key	2006 [48]	NART	11	27.65	16.8	109	11.1
Thompson	1993 [49]	NART	10	25.8		115.8	NR
WAIS studies:							
Pendleton-Jones	1991 [38]	WAIS	30	24.4	59% ideal	104.4	12.6
Mathias	1998 [32]	WAIS-R	34	22	15.3	97.3	16.3
Galderisi	2003 [50]	WAIS	14	Approximately 23.7	15.4	100	10.8
Holliday	2005 [51]	WAIS-R	47	26.3	17.9	111.3	7.6
Connan	2006 [52]	WAIS-R	16	25.4	16.2	117.6	16.7
Steinglass	2006 [53]	WASI	15	25.6	19	109.8	12.1
McCormick	2008 [54]	WAIS	16	Approximately 25.2	NR	96.5	12.9
Dura	1989 [10]	WAIS-R	20	14.7	NR	102.5	NR
McDowell	2003 [55]	WAIS-R	98	27.2	15.9	98.2	12.1
Grunwald	2001 [56,57]	HAWIK	10	15.9	15.2	115.2	8
Gordon	1984 [58]	WAIS and WISC-R	10	15.7	NR	112.7	13.1
Grunwald	2002 [59]	HAWIK	16	15.3	14.8	113.5	12.4
Bayless	2002 [60]	WAIS	59	24.3	16.8	103.6	12.1
Wilbur	1981 [61]	WAIS/WISC	34	17	NR	111	NR
Ranseen	1992 [62]	WAIS-R	38	21.7	NR	99.5	16.6
Small	1982 [63]	WAIS	27	20.6	NR	108.1	10.4

BMI = Body Mass Index; HAWIK = Hamburg-Wechsler-Intelligenztest für Kinder (German version of WAIS); NART(-R) = National Adult Reading Test (Revised); NR = not reported; SD = standard deviation; WAIS(-R) = Wechsler Adult Intelligence Scale (Revised); WASI = Wechsler Abbreviated Scale of Intelligence (short form of WAIS); WISC(-R) = Wechsler Children Intelligence Scale (Revised).

### Estimated IQ in AN as measured by the NART

The NART was used in 14 studies, with a total sample size of 365 AN patients. The sample size of the trials ranged between 10 and 46. The mean IQ of the 14 studies ranged from 96.1 to 116.8. A meta-analysis using a random effects model revealed an estimate of the mean standardised mean difference (SMD) of 0.72 with a 95% confidence interval of 0.53 and 0.91.

**Figure 3:**
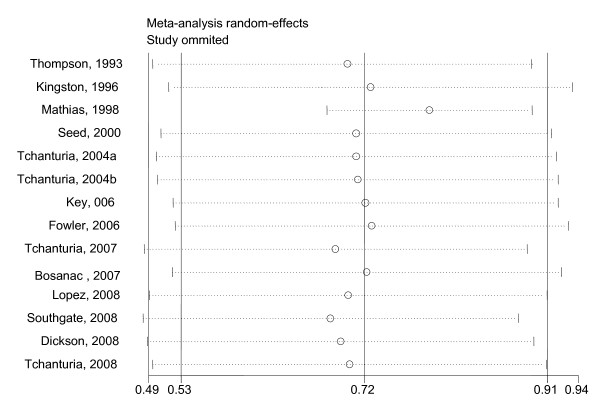
**Results of an influence analysis in which the meta-analysis is re-estimated omitting each study in turn**. Rerunning the analysis without the study by Mathias and Kent [32] increased the standardised mean difference (SMD) from 0.72 to 0.79 (95% CI 0.68 to 0.90). The straight vertical lines at 0.72, 0.53 and 0.91 represent the SMD and upper and lower 95% confidence intervals of the complete study analysis.

The SMD of 0.72 means that patients with EDs score on average 10.8 units (95% CI 7.9 to 13.6) above the average IQ of the normative population. There was evidence of considerable heterogeneity across studies (Cochran's Q test: X^2^_(13) _= 43.7, *p *< 0.001 and I^2 ^= 70.2%). This variance was particularly due to the data from Mathias and Kent [32]. In this study, the AN group obtained lower IQ compared with norms, with an effect size of *d *= -0.26 (see Figure 1). To investigate the influence of this study on the overall meta-analysis, the meta-analysis was repeated excluding one study at a time to ensure that the results were not biased by a single outlier. Rerunning the analysis without the Mathias and Kent study [32] increased the SMD slightly from 0.72 to 0.79 (95% CI 0.68 to 0.90). There was no more evidence for heterogeneity between studies (Cochran's Q test: X^2^_(12) _= 9.58, p = 0.65, I^2 ^= 0%). The influence of other studies on the overall estimate was minor (see Figure 3).

### Publication bias

A funnel plot based on all 14 studies did not indicate publication bias either with or without Mathias and Kent [32], nor did formal tests (Begg's test *z *= 0.47, *p *= 0.64 and Egger's test t = 0.86, *p *= 0.40 and Begg's test *z *= 0.63, *p *= 0.53 and Egger's test t = 0.69, *p *= 0.50, respectively). The trim and fill method did not indicate missing studies.

In summary, there is no evidence for publication bias and the estimated effect size found from the random effects model is realistic. The overall conclusion from this analysis is that people with AN tend to consistently score higher than population norms on the NART across published studies.

### IQ in AN as measured by Wechsler's tests

Wechsler's tests were used in 16 studies with a total sample size of 484 patients. The WAIS was used in five studies, WAIS-R in six studies, the short form of the WAIS (WASI) in one study, two studies used both the WAIS and the WISC according to the age of participants and two studies used HAWIK (the German version of WAIS). The sample size of the trials ranged between 10 and 98. The mean IQ of the 16 studies ranged from 96.5 to 117.6. Using a random effects meta-analysis, the estimate of the pooled SMD was a small to medium effect size of *d *= 0.40 (95% CI 0.18 to 0.61) across all studies. The SMD of 0.40 translates that patients with EDs score on average 5.9 units (95% CI 2.7 to 9.2) above the average IQ of the normative population.

The meta-analysis (see Figure 2) revealed a high degree of heterogeneity across the studies (X^2^_(15) _= 81.2, *p *< 0.001), with an index of inconsistency of 81.5%; parameters that justified the use of a random effects model. The heterogeneity was not due to a single study, as when the meta-analysis was re-estimated omitting each study in turn, no single study had a significant influence on the results. About half of the included studies showed a moderate to high effect size, whereas the remaining showed little or no mean effect, which may explain this heterogeneity. This observation will be discussed later in this section.

The analyses were repeated excluding one study at a time to investigate the influence of each individual study on the overall meta-analysis summary.

**Figure 4:**
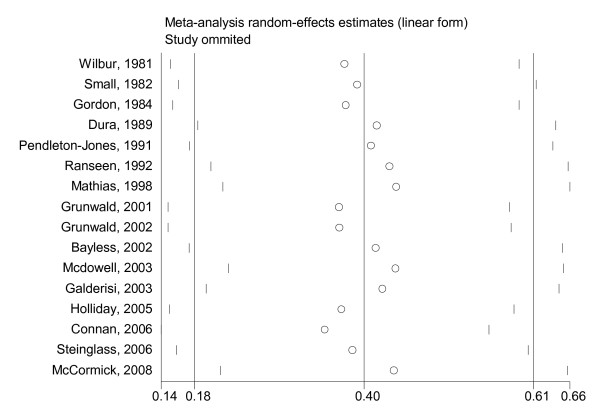
**Results of an influence analysis in which the meta-analysis is re-estimated omitting each study in turn**. The straight vertical lines at 0.40, 0.18 and 0.61 represent the estimated standardised mean difference (SMD) and upper and lower 95% confidence intervals of the complete study analysis.

The influence of individual studies on the estimated overall effect size (removing each study and recalculating overall effect) was minor. Rerunning the meta-analysis excluding one study at a time resulted only in minor differences of the estimated SMDs (range of *d *= 0.35 to 0.44; see Figure 4).

### Publication bias

**Figure 5:**
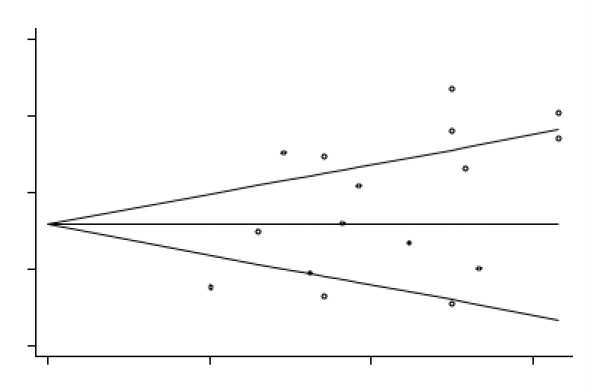
**Begg's funnel plot with pseudo-95% confidence limits for meta-analysis based on Wechsler studies**.

From the Forest plot (Figure 2), it could be assumed that there are two types of studies: those with an ES around 0 (no differences between the IQ of those with AN and norms) and those with an ES around 0.75 (moderate to high differences between populations). This, alongside the fact that the funnel plot based on all 16 studies is slightly asymmetrical, would suggest some publication bias towards studies with larger effect sizes. This was confirmed by Egger's test (Egger's test t = 2.11, *p *= 0.05, Begg's test *z *= 1.09, *p *= 0.27). However, the trim and fill method did not estimate any missing study, which suggests that the results are robust (Figure 5). Also, studies with an ES around 0 or 0.75 seem not to be different in terms of the year of the study, the number, age or BMI of participants, comorbidity, or whether participants were in/outpatients. Therefore, the results suggest the absence of an identifiable publication bias.

### IQ and BMI

BMI data available from the studies included in this review were correlated with the estimated IQ obtained from studies in order to understand the role of nutritional status in the intellectual functioning of people with AN.

Firstly, a correlation analysis was run between BMI and IQ with all data available (from NART or Weschler's scales). The results did not show a correlation between these two variables. However, when separating studies according to the instrument used to estimate IQ (Weschler's scales or NART), a trend for a significant moderate correlation was found in those studies using the NART (Spearman coefficient = 0.56, *p *= 0.07), meaning that taking all these studies as a group, lower premorbid IQ was associated with lower BMI. No correlation was found between IQ estimated by Weschler's scales and BMI, suggesting that current IQ does not fluctuate with changes in BMI.

### IQ and recovery

We were able to identify only four studies including women with past history of AN now recovered (N = 64, see Table 2). Three of these studies used the NART and the revised NART (NART-R) and only one study used the WAIS. The small number of studies did not allow a formal random effect meta-analysis. However, in all four studies, patients scored significantly more than 100, as the 95% confidence intervals show (Table 2).

**Table 2:** Studies in recovered anorexia nervosa samples

Lead author	Publication date and reference	IQ test	N	IQ mean (95% CI)	IQ SD
Tchanturia	2004 [37]	NART-R	18	113.6 (109.1 to 118.1)	9.1
Tchanturia	2007 [36]	NART	14	118.1 (114.8 to 121.4)	5.7
Bosanac	2007 [35]	NART	12	114.5 (110.9 to 118.1)	5.7
Pendleton-Jones	1991 [38]	WAIS	20	109.3 (102.0 to 116.6)	15.5

SD = standard deviation.

A descriptive comparison of these results suggests that those recovered from AN score higher on IQ measures (mean IQ ranged from 109.3 to 118.1) than groups with current AN (mean IQ in current AN ranged from 96.1 to 116.8 and 96.5 to 117.6 using the NART and WAIS, respectively) and norms. Along the same lines as the conclusion above, this very preliminary result may indicate that those who recover tend to have higher premorbid IQ. More research in recovered samples is needed in order to clarify this observation.

## Discussion

The aim of this study was to provide a comprehensive systematic review of the literature including a meta-analysis for estimated IQ data in AN compared with the average IQ obtained from normative data. Studies included in this review were selected on the basis of quality of the data and validity of instruments used to estimate intellectual ability. Thus, NART and Weschler's scales were selected as the most common and reliable measures.

It was established that most people with AN have higher average IQ scores compared with the average of normative data. Specifically, studies using the NART consistently showed a higher IQ in AN patients in comparison with established norms. However, those using Wechsler's scales obtained more heterogeneous results, with half of the studies showing moderate to high effect sizes (average ES = 0.75) and half of them with low or negligible effect sizes (average ES around 0). Examination of the characteristics of the various studies (for example, age at testing, BMI, reported comorbid conditions, and so on) was not able to explain this heterogeneity. These results overall show that people with AN have at least as high IQ as norms, which indicates a difference, compared to other psychiatric conditions.

There are two points that are worth highlighting in this discussion about the heterogeneity of results. Although the WAIS and NART are highly correlated, the NART estimates premorbid IQ and Wechsler's scales measure current ability. Predictions could be made from NART scores about performance, verbal and total IQ based predominantly on verbal abilities, which are thought to be generally preserved in AN [1,7], whereas Weschler's scales assess mixed verbal, performance and visual spatial abilities. The latter have been reported to be more impaired in people with acute AN (see, for example, [7]). It might be possible that those studies using Wechsler's scales with lower effect sizes are demonstrating differences in the performance of the samples that are not evident in full scale IQ data (for example, differences in performance versus verbal IQ or lower scores in scales involving visual spatial abilities). We are not able to clarify this point with this review, as most studies provided full scale IQ only, as previously mentioned. Also, Wechsler's scales used in the studies included in this review are composed of different scales (for example, WAIS, WISC, short version, and so on) and there was no consistent use of one single instrument.

Both premorbid and current IQ yield valid and interesting information for future studies and the measure selected will depend on the nature and objectives of future studies. Therefore, it seems that simple and reliable measures such as the NART provide more consistent data on IQ, without the bias that anomalies in cognitive performance (typically present in the acute phase of AN) may introduce in test performance. Also, from the available literature, it is still hard to draw firm conclusions regarding performance and verbal IQ. Clear reporting in future studies (for example, separating verbal from performance IQ) will help to address this point.

A coordinated approach and consensus of IQ measures in the field will make data more comparable and will provide better insight into the relationship between illness severity and the neuropsychological profile of AN. For example, meta-analyses and systematic reviews conducted in schizophrenia allowed researchers to identify that, before the onset of psychosis, IQ scores are approximately 0.5 standard deviations below that of healthy comparison subjects, and low IQ could be considered as one of the risk factors for schizophrenia [33,34].

In order to explore the question about the potential contribution of IQ as a predictor of recovery, we made an attempt to review studies on recovered AN populations. Only four studies [35-38] reported IQ data on women with a past history of AN. We did not have efficient power to draw strong conclusions from the available studies. However, a meta-analysis showed that people who had recovered from the illness had higher IQ in comparison to norms and studies on acute AN groups, included in this paper. There is a growing literature suggesting that IQ level can predict treatment outcome in psychiatric conditions such as schizophrenia and autism (see, for example, [39]). We predict that this line of research will also be highly informative for the ED field. For example, it was reported that higher IQ predicts completion of psychological treatment in early studies [14]. To our knowledge, none of the treatment studies conducted in EDs looked at drop-out data in the context of IQ. In general, it is clear that all treatment studies in AN have high drop-out rates [40]. From the results of this study, it seems likely that successfully treated patients with AN have higher premorbid IQ, which would support the hypothesis of the higher the IQ, the better the treatment prognosis. However, more studies will be needed to confirm this prediction and which other factors may be involved (for example, environmental, physiological, and so on).

We believe that this systematic appraisal of the literature was helpful in highlighting a trend that suggests that people with AN have average or higher than average IQ (in both NART and WAIS studies). The benefit of this appraisal of current knowledge will help researchers in planning future studies and formulate important questions, such as: do patients with higher IQ have better prognosis? How could high IQ be effectively used in psychological treatment? Is IQ decline evident in AN? Whereas the most obvious reason will be malnutrition, none of the studies so far have used premorbid and current IQ measures simultaneously.

Finally, this study helped us to reflect on the fact that comparison clinical groups should be carefully selected in future studies on AN, because IQ will be an important contributing factor in social cognition, cognitive tasks, either using self-report or experimental instruments.

This review has some limitations. Firstly, one of the limitations is the retrospective nature of the data. Secondly, results in studies using Wechsler's scales showed high heterogeneity, which makes it difficult to draw strong conclusions from these scales. We examined the influence of the use of different versions of the test as well as different age groups and other clinical characteristics of the samples. Despite efforts to clarify the reasons behind such different results, we were not able to identify a consistent factor across studies. We have also confirmed a lack of literature related to other ED diagnostic categories, such as BN, EDNOS or recovered ED groups in the context of IQ. This fact precludes the possibility to generalise these results to other ED groups and more importantly, the examination of potential relationships between IQ, recovery and prognosis. Thirdly, it is important to mention that most of reviewed studies involved clinical participants (inpatients or outpatients) or registered volunteers. One of the possibilities is that these samples are highly selected because they are not population-based samples, rather people who seek treatment in the clinics or are willing to participate in research. This may relate to higher education and IQ performance but these questions are beyond the scope of this study. Finally, as the main outcome of most of studies included in this review was not IQ performance, it is likely that a publication bias exists, however it is not possible to address it in this study.

From this review some recommendations arise: the majority of accessed studies used the NART or Wechsler's scales. It will be useful if future studies continue to use these measures to make future data comparable. It will also be desirable for all studies looking at neuropsychological factor to include an IQ measure due to the effect that it may have on neuropsychological task performance. Finally, since neuropsychological research is increasing, it will be helpful to report covariate analysis in relation to IQ; for example, with treatment outcomes, symptom severity and recovery.

## Conclusions

This research highlights an important and underexamined factor in AN. The main conclusion is that IQ in AN is at least as high as the average IQ found in the normative population and most studies show that this group have a high average IQ. There is a preliminary but important observation about IQ in the recovered population, which is that this group may represent a group with higher IQ than norms and current AN groups, opening the question about the influence of this factor on treatment and recovery. We think that exploring IQ in the context of treatment and recovery may provide useful information for clinicians and researchers.

## Competing interests

The authors declare that they have no competing interests.

## Authors' contributions

CL contributed to the design of the study, performed the main search, data extraction, data synthesis, supported general analyses and interpretation of data, and contributed to drafting the manuscript. DS contributed to the design of the study, performed the statistical analyses, drafted the results section and critically reviewed the manuscript. KT contributed to the design of the study, did an independent search of the papers, supervision/interpretation of data and drafted the main part of the manuscript. All authors approved the final manuscript.
